# Anti-Inflammatory and Antioxidant Activities of* Salvia fruticosa*: An HPLC Determination of Phenolic Contents

**DOI:** 10.1155/2016/7178105

**Published:** 2016-01-03

**Authors:** Rima Boukhary, Karim Raafat, Asser I. Ghoneim, Maha Aboul-Ela, Abdalla El-Lakany

**Affiliations:** ^1^Department of Pharmaceutical Sciences, Faculty of Pharmacy, Beirut Arab University, Beirut 115020, Lebanon; ^2^Department of Pharmacology and Therapeutics, Faculty of Pharmacy, Beirut Arab University, Beirut 115020, Lebanon; ^3^Department of Pharmacology and Toxicology, Faculty of Pharmacy, Damanhour University, Damanhour 22514, Egypt

## Abstract

*Objectives*. *Salvia fruticosa* Mill. (*S. fruticosa*) is widely used in folk medicine. Accordingly, the present study was designed to evaluate the antioxidant and anti-inflammatory activities of* S. fruticosa*, and to determine the phenolic constituents of its extracts.* Methods*. The antioxidant activity was determined using 2,2-diphenylpicrylhydrazyl assay. Total phenolic contents were estimated using Folin-Ciocalteu reagent, and high-performance liquid chromatography was performed to identify phenolic constituents. To evaluate the anti-inflammatory activity, carrageenan-induced mouse paw edema was determined plethysmographically.* Key Findings*. Different plant extracts demonstrated strong radical scavenging activity, where the ethyl acetate extract had the highest value in the roots and the lowest in the aerial parts. This antioxidant activity was correlated to the total phenolic content of different extracts, where rutin and luteolin were the most abundant constituents. Interestingly, both the roots and aerial parts revealed a significant anti-inflammatory activity comparable to diclofenac.* Conclusions*. This study is the first to demonstrate pharmacologic evidence of the potential anti-inflammatory activity of* S. fruticosa*. This activity may partly be due to the radical scavenging effects of its polyphenolic contents. These findings warrant the popular use of the East Mediterranean sage and highlight the potential of its active constituents in the development of new anti-inflammatory drugs.

## 1. Introduction

Oxygen is essential for the life of all aerobic organisms; nevertheless, oxidative stress may induce damage to cellular biomolecules and is implicated in many diseases [1]. On the other hand, phenolic compounds derived from plants have received high attention during recent years owing to their potential antioxidant effects and positive influence on human health. Plant phenolics also interact to prevent various diseases by quenching oxygen-reactive free radicals [2–4].

The use of plants as remedies is as ancient as human civilization, and plants still remain the main sources of useful drugs. Crude plant extracts have been recently documented worldwide as an important source of phytochemicals having several biological activities, including antioxidant and anti-inflammatory effects [2, 3, 5, 6]. The genus* Salvia*, as one of the largest genera of the family Lamiaceae (Labiatae), is represented by about 900 species throughout the world. Numerous species of the* Salvia* genus are economically important, have been used since ancient times in folk medicine, and have also been subjected to extensive pharmacognostic research to identify biologically active constituents [7]. Particularly, the species* Salvia fruticosa* Miller (*S. fruticosa *Mill.), also named as* S. libanotica* and formerly named as* S. triloba*, is native to the eastern Mediterranean and is known as East Mediterranean sage or Lebanese sage [8, 9]. It represents most of the imported sage in the United States rather than* S. officinalis*. In folk traditional medicine,* S. fruticosa* is widely used by people, herbalists, and pharmacists, either internally as infusions, inhaled in steam baths, or even applied externally. The plant is boiled as a tea for the relief of different pains, colds, influenza, and many other disorders. This endemic Lebanese plant has also been used for improving memory and as hypoglycemic agent with an antioxidant potential [5, 8, 9]. Indeed, the Lamiaceae family includes a large number of plants that are well known for their antioxidant properties. In particular, the* genus Salvia* has been subject of intensive study in the past decades for its antioxidative and anti-inflammatory effects in relation to the active constituents, including the phenolic contents [6, 10, 11]. The anti-inflammatory activity of phenolics and flavonoids has also been shown to be a result of their antioxidant effect [11, 12]. Therefore, this research aimed to evaluate the anti-inflammatory and antioxidant activities of* S. fruticosa* root and aerial extracts, and to relate these activities to the plant extract phenolic constituents.

## 2. Materials and Methods

### 2.1. Plant Material

Fresh* S. fruticosa* Mill. (synonyms:* S. libanotica* and* S. triloba*) was collected during the flowering period in March and April 2012 from southern Mediterranean region at altitudes ranging from 200 to 400 m at the littoral of Beirut, Lebanon. The plant was identified by Dr. Georges Tohme, Professor of Taxonomy. A dried specimen was kept at the Faculty of Pharmacy, Beirut Arab University (herbarium number: ps-14-12). The plant was dried under shade at 25°C and the dried aerial parts and roots were ground separately with a blender.

### 2.2. Preparation of Plant Extract

The powdered parts of plant (80 g) each were extracted successively with chloroform, ethyl acetate, methanol, and butanol for 24 hours. The residues were removed by filtration. The extracts were concentrated (yield about 7%) in a rotary evaporator under reduced pressure at a temperature of 40–50°C and then lyophilized to get powders.

### 2.3. Determination of DPPH Scavenging Activity

*In vitro *2,2-diphenyl picryl hydrazyl (DPPH) radical scavenging activity was carried out by adopting the method of Blois [13]. The hydrogen atoms or electrons donation ability of the corresponding extracts was measured from the bleaching of purple colored methanol solution of stable free radical DPPH [14]. The absorbance of different extracts was read against a blank at 517 nm at different time intervals for duration of 60-minute incubation period at room temperature. Lower absorbance of the reaction mixture indicated higher free radical scavenging activity. Vitamin C was used as a positive control showing 100% scavenging activity.

DPPH radical scavenging activity %  (SC%) = (*A*_*c*_ − *A*_*I*_/*A*_*c*_)*∗*100, where *A*_*c*_ is the absorbance of control and *A*_*I*_ is the absorbance of extract.

### 2.4. Determination of Phenolic Compounds

Total phenolic compounds were determined using Folin-Ciocalteu reagent (FCR) as described by Slinkard and Singleton [15]. In brief, serial dilutions of* S. fruticosa* roots and whole aerial parts were prepared. 0.2 mL of each solution was added to a glass test tube and 1 mL of FCR, 0.8 mL of Na_2_CO_3_ (7.5%) were pipetted to it. The preparations were stored at room temperature for 60 minutes and then their absorbances were read at 765 nm. Absorbance values were compared with standard solution of gallic acid equivalent [16]. Also, the quantities of HPLC-identified phenolic contents (gallic acid, rutin, and luteolin) were determined by carrying out column chromatography and determining the weight of corresponding fractions.

### 2.5. HPLC Analysis

A sample (200 *μ*g/mL) was prepared in methanol. It was homogenized by using a vortex. Then, the plant extract was passed through a 0.45 *μ*m filter before injection into a reverse phase NOVA-PAK C18 column at ambient temperature (20°C). The mobile phase was methanol and phosphate buffer (43 : 57). The flow rate was 1.0 mL/min and the wave length of detection was set at 254 nm [17].

### 2.6. Evaluation of the* In Vivo* Anti-Inflammatory Activity

The carrageenan-induced mouse paw edema model was adopted as recently described by our group [18]. Briefly, the tested methanolic extracts were administered intraperitoneally (i.p.) at a dose of 200 mg/kg body weight. The volume of the carrageenan-injected paw was determined plethysmographically immediately after injection and 4 hours later. The difference between the two readings gave the actual edema volume increase to calculate the percentage protection. The anti-inflammatory activity of the tested extracts relative to that of diclofenac was also calculated.

### 2.7. Animals

Male albino mice were kept for 1 week prior to the experimentation at the animal house of the Faculty of Pharmacy, Beirut Arab University. The environment consisted of a temperature of 25 ± 1°C, and standard mouse cages with a 12 h light/dark cycle. The animals had free access to water and standard laboratory chow (20% proteins, 5% fats, and 1% multivitamins) [2, 19]. Animal care and handling for the research were performed in accordance with the regulations and guidelines stipulated by the Institutional Animal Care and Use Guidelines (IACUG) at Beirut Arab University, Lebanon (IRB approval code: 2015A-020-P-P-0055).

### 2.8. Statistical Analysis

Data were statistically analyzed using one-way analysis of variance (ANOVA) followed by Tukey simultaneous comparison *t*-values. Statistical differences were considered to be significant at *p* < 0.05.

## 3. Results

**Figure 1:**
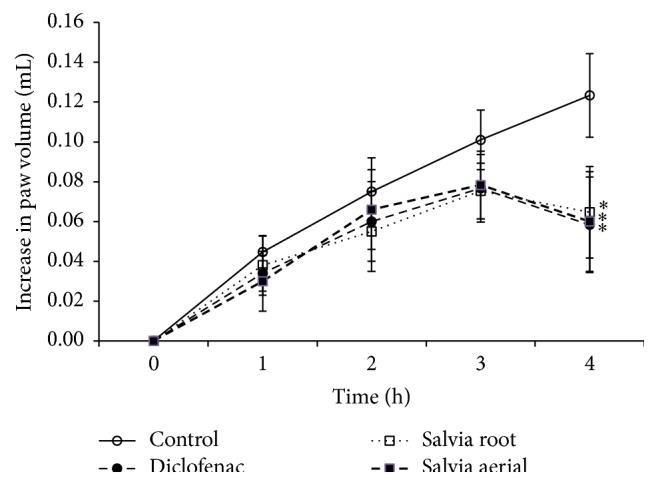
Effects of* S. fruticosa* extracts on mouse hind paw edema. The actual edema volume increase was measured relative to that of standard drug diclofenac 50 mg/kg. Values are presented as means ± SD, *n* = 4–7. *∗* denotes significant difference from control value at *P* < 0.05.

The anti-inflammatory activity of the plant was carried out* in vivo* and the results were shown in Figure 1. Both the aerial parts and the roots of the crude extracts exhibited significant protection at 4 h against carrageenan-induced mouse paw edema by 50% and 44%, respectively. The promising anti-inflammatory activities of the aerial parts and the roots extracts relative to that of the standard drug diclofenac were 0.9 and 0.8, respectively.

Also, the* in vitro* antioxidant activity by using DPPH was investigated. The free radical scavenging activity of both aerial and root extracts is presented in Table 1. As can be seen, the ethyl acetate root extract of* S. fruticosa* showed highest scavenging activity at 60 min (42.15 ± 2.06) as compared to that of aerial parts being the lowest (32.20 ± 4.50). The root extracts of methanol, chloroform, and butanol showed their scavenging activities in the decreasing order. However, the aerial parts extracts of butanol, methanol, and chloroform showed their scavenging activities in the increasing order (Table 1). The root extract showed the lowest scavenging activity at 60 min for butanol extract (25.43 ± 2.60), in comparison to that of aerial parts, where chloroform extract showed the highest one (41.59 ± 6.10).

**Figure 2:**
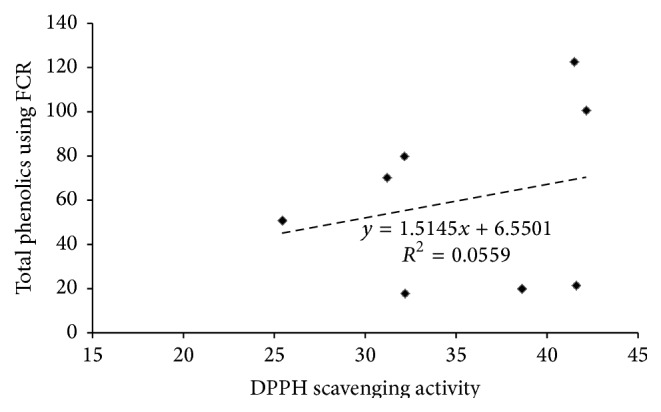
Relationship between antioxidant activity and total phenolic contents of different* S. fruticosa* extracts. Correlation coefficient *R* ^2^ = 0.0559 (*r* = 0.24).

In addition, the total phenolic contents of different extract solutions were determined by performing a reaction with FCR. Results were compared with the standard solutions of gallic acid equivalents and were presented in Table 2. The ethyl acetate extract showed highest FCR absorbance (100.66 ± 3.34) in the roots as compared to highest absorbance of methanol extract in aerial parts (122.67 ± 0.44) as shown in Table 2. Regarding the corresponding antioxidant activity values at 60 min, in comparison with the phenolic contents, it could be noticed that the highest scavenging activity of ethyl acetate root extract (42.15 ± 2.06) corresponds to highest phenolic content (100.66 ± 3.34). On the other hand, the lowest scavenging activity in the ethyl acetate extract of the aerial parts (32.20 ± 4.50) corresponds to lowest phenolic contents (17.89 ± 0.24). This observation demonstrated that there is certain correlation between the free radical scavenging activity and the total phenolic contents of the plant extracts. Indeed, an overall positive correlation between antioxidant activity and the total phenolic content of the extracts was illustrated in Figure 2. The relationship between the whole antioxidant activities and total phenolic contents of all the tested extracts based on the correlation (linear relationship) displayed a weak positive correlation (*R* ^2^ = 0.0559; *r* = 0.24).

Moreover, concerning the relationship with anti-inflammatory activity that both the aerial and root extracts showed promising effects (50% and 44%, resp.), the following was noticed. A similarity was evident in case of the significant radical scavenging activity ranging in the aerial parts from 32.20 to 41.59 and in the roots from 25.43 to 42.15. Nevertheless, this seemed somewhat different from the lower total phenolic content of all added aerial extracts (182.06 mg gallic acid/g extract) compared to that of twofold higher content in all added root extracts (301.76 mg gallic acid/g extract).

**Figure 3:**
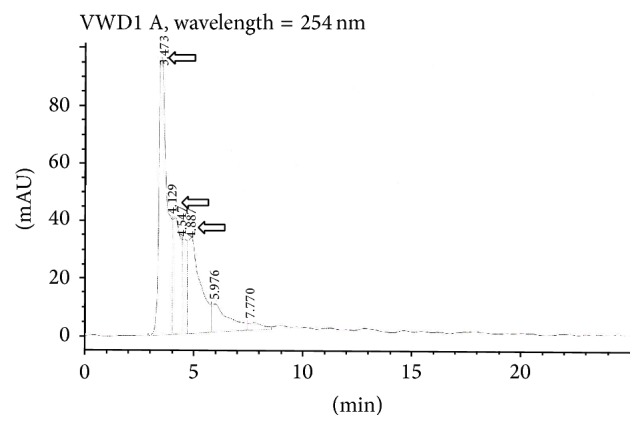
HPLC chromatogram for the determination of phenolic constituents of* S. fruticosa* extract. The methanolic plant extract showed several flavonoids. Major constituents, luteolin and rutin, are represented by arrows at the retention time values of 3.473 min and 4.887 min, respectively. Gallic acid also appeared at 4.129.

Furthermore, a column chromatography of the plant extracts isolated a high yield of phenolic contents, such as gallic acid (37 mg), rutin (23 mg), and luteolin (9.23 mg), as also identified in the HPLC analysis (Figure 3). The analysis of whole plant extracts showed that a large number of flavonoids were present. Luteolin and rutin were the most abundant phenolic constituents and were readily identified by comparison with authentic standards as shown in Figure 3. Luteolin appeared first at the retention time value of 3.473 and then rutin at 4.887. Gallic acid also appeared at 4.129.

## 4. Discussion

*S. fruticosa* was found to be a rich source of antioxidants, where the ethyl acetate root extract had the highest radical scavenging activity and the highest concentration of phenolics. In addition, the aerial parts of the plant showed the higher anti-inflammatory activity. Both the aerial parts and the roots exhibited significant protection against carrageenan-induced mouse paw edema with promising activities of the two crude extracts relative to that of the standard drug diclofenac reaching 90%. The anti-inflammatory activity of the extracts was in line with their antioxidant activity better than their phenolic contents. The observed anti-inflammatory activities of this extract may partly be attributed to the overall effects of the phenolics and other plant constituents having potent anti-inflammatory actions similar to diclofenac. The extent of decrease in the absorbance of DPPH in the presence of antioxidants correlates with their free radical scavenging potential. Accordingly, the ethyl acetate root extract of* S. fruticosa* showed highest scavenging activity as compared to that of aerial parts. The methanol, chloroform, and butanol root extracts showed lower activities in decreasing order. However, other studies carried out on several species such as* Salvia verticillata* showed the highest scavenging activity in methanol extract [20]. This activity was attributed to the presence of phenolic constituents present in this plant in different proportions and identified as gallic acid and rosmarinic acid which occurred in higher amount and was identified by TLC screening compared with references [20]. Concerning total phenolic content, the ethyl acetate root extract showed highest FCR absorbance as that of methanol extract of aerial parts meaning that they contained a high concentration of phenolic contents such as gallic acid (root), rutin, and luteolin (aerial) that were isolated by column chromatography and identified by HPLC. Although the ethyl acetate extract showed highest activity in the roots, it demonstrated lowest one in the aerial parts. In addition, this highest scavenging activity of ethyl acetate root extract corresponded to highest phenolic contents. In parallel, the lowest scavenging activity in the ethyl acetate extract of the aerial parts corresponded to lowest phenolic contents demonstrating that there was certain correlation between the free radical scavenging activity and the total phenolic contents of the different plant extracts. The overall positive correlation (*r* = 0.24) between antioxidant activity and the total phenolic content of the extracts might link the phenolic contents of this plant to its antioxidant action. This finding may warrant the popular use of* S. fruticosa* worldwide for treating several illnesses. Indeed, the antioxidant activity is considered one of the important bioactivities of* Salvia* plants, and it is attributed mainly to the major effective content of polyphenols and terpenes [6, 7]. Some polyphenolic extracts from* Salvia* were examined for antioxidant activity in correlation with their polyphenolic content. Some of the results revealed that polyphenolic extracts had strong free radical scavenging activity against DPPH. Alternatively, other results showed that the total polyphenolic content is not correlated with antioxidant activity in other extracts [7]. Regarding the anti-inflammatory activity, both aerial and root extracts showed promising activities in line with that of free radical scavenging ones in the aerial parts and in the roots. However, it seemed somewhat different from the cumulative lower total phenolic content of different aerial extracts and that of higher by twofold total content in the root extracts. Therefore, total phenolic contents may at least in part be responsible for the radical scavenging and anti-inflammatory activities of* S. fruticosa*. Similarly,* S. miltiorrhiza* contains both hydrophobic and hydrophilic compounds. More than 30 diterpenes have been isolated and identified from the hydrophobic fraction. Investigations on hydrophilic compounds revealed major constituents including water-soluble phenolic acids [11]. Indeed, several researches on* Salvia* plant extracts displayed high antioxidant and anti-inflammatory activities but contained low levels of phenolics and flavonoids. Then, the potential anti-inflammatory activities of the plant extracts may at least in part be due to the radical scavenging activity of their polyphenolic content, in accordance with other previous researches and findings [6, 7, 21, 22]. The depiction of correlations (linear relationship) between extracts activity and polyphenolic contents helps to better understand the relationship between different activities and major extract constituents. A good agreement with literature reports was evident in that polyphenolics are the major antioxidant compounds in medicinal plants [6]. However, it should be noted that, sometimes, the antioxidant activities of some other plants did not show a good correlation with their polyphenol contents. These observations demonstrate that, in addition to polyphenols, other constituents may contribute to the antioxidant activities of medicinal plants, such as trace metal contents [6]. In many herbs, flavonoids containing multiple hydroxyl groups have strong antioxidant effect [3, 4, 23]. The HPLC of whole* S. fruticosa* extract also showed that a large number of flavonoids were present. Luteolin and rutin were the most abundant phenolic constituents. These phenolic compounds and others have also been successfully isolated from other* Salvia* species [10, 17, 20, 22, 23].

## 5. Conclusions

To our knowledge, the first evidence of powerful anti-inflammatory activity of* S. fruticosa* was demonstrated by this novel research.* S. fruticosa* was also found to be a rich source of antioxidants, where the root extracts had highest antioxidant activity and concentration of total phenolics. The potential anti-inflammatory activity of the plant extracts may hence be, at least in part, due to the radical scavenging activity of their polyphenolic content. Therefore, further evaluation of the anti-inflammatory activity of different* Salvia* extract fractions and isolated active constituents is warranted. Actually, the above-mentioned promising pharmacological activities highlight the plant's potential use in the development of new anti-inflammatory drugs. This investigation should be encouraged given the wide distribution, ease of cultivation, and the popular worldwide use of the East Mediterranean sage plant.

## Figures and Tables

**Table 1:** Free radical scavenging activity of *Salvia fruticosa *extracts at different time intervals (min).

	Sample	At 0′	At 20′	At 40′	At 60′
Aerial parts	Chloroform extract	0.01 ± 0.01	29.00 ± 12.60^*∗*^	35.65 ± 4.40^*∗*^	41.59 ± 6.10^*∗*^
	Methanol extract	0.01 ± 0.01	29.96 ± 4.20^*∗*^	36.69 ± 6.40^*∗*^	41.50 ± 6.70^*∗*^
	Ethyl acetate extract	0.01 ± 0.01	17.78 ± 6.60^*∗*^	24.40 ± 2.05^*∗*^	32.20± 4.50^*∗*^
	Butanol extract	0.01 ± 0.01	19.81± 2.06^*∗*^	30.70 ± 6.50^*∗*^	38.62 ± 8.00^*∗*^

Roots	Chloroform extract	0.01 ± 0.01	11.72 ± 2.04^*∗*^	13.33 ± 2.10^*∗*^	31.2 ± 2.09^*∗*^
	Methanol extract	0.01 ± 0.01	13.42 ± 2.03^*∗*^	19.33 ± 4.30^*∗*^	32.16 ± 4.20^*∗*^
	Ethyl acetate extract	0.01 ± 0.01	20.46 ± 4.04^*∗*^	28.39 ± 2.03^*∗*^	42.15 ± 2.06^*∗*^
	Butanol extract	0.01 ± 0.01	13.05 ± 2.10^*∗*^	20.47 ± 2.20^*∗*^	25.43 ± 2.60^*∗*^

Values are presented as % scavenging activity means ± SD. *∗* denotes significant difference from corresponding value at 0′. The level of significance was set at *P* < 0.05. Data were analyzed using one factor ANOVA followed by Tukey simultaneous comparison *t*-values.

**Table 2:** Free radical scavenging activity (SC%) of* Salvia fruticosa* extracts versus their total phenolic content.

Extract	Free radical scavenging activity at 60 min	Total phenolics as mg gallic acid/g extract
	(mean SC% ± SD)	(mean ± SD)
Aerial parts		
Chloroform extract	41.59 ± 6.10	21.50 ± 0.38
Methanol extract	41.50 ± 6.70	122.67 ± 0.44
Ethyl acetate extract	32.20 ± 4.50	17.89 ± 0.24
Butanol extract	38.62 ± 8.00	20.0 ± 0.40
Roots		
Chloroform extract	31.2 ± 2.09	70.3 ± 0.9
Methanol extract	32.16 ± 4.20	80 ± 1.68
Ethyl acetate extract	42.15 ± 2.06	100.66 ± 3.34
Butanol extract	25.43 ± 2.60	50.80 ± 0.34
